# Simulating Error Due to Acquired Thermoelectric Inhomogeneity

**DOI:** 10.3390/s24165256

**Published:** 2024-08-14

**Authors:** Zida Li, Jingliang Chen

**Affiliations:** 1 Detroit Green Technology Institute, Hubei University of Technology, Wuhan 430068, China; zidli2021@gmail.com; 2 School of Computer Science, Hubei University of Technology, Wuhan 430068, China

**Keywords:** thermocouple, temperature measurement error, thermoelectric inhomogeneity, thermocouple with controlled temperature field

## Abstract

The best method to prevent error due to inhomogeneity is to use a new thermocouple design—the thermocouple with controlled temperature field (TCTF). It uses the auxiliary furnace to control the temperature field along its legs. Such a design allows setting and maintaining the temperature field along the thermocouple (TC) legs for the sensor. Error due to inhomogeneity of TCs cannot appear in a stable temperature field. However, the auxiliary furnace and TCs, to control the temperature field, have errors, so the temperature field along the main TC is maintained with some error. This leads to residual error due to acquired inhomogeneity of the TCTF. We constructed the mathematical models to fit the experimental data of error due to drift for the type K TC. The authors used the constructed models to study error due to inhomogeneity of the TCTF and the conventional type K TC under considerable changes in temperature field. The main results of modelling are as follows: (i) if the changes in temperature field exceed 7 °C, error due to inhomogeneity of the TCTF is lesser than that of the conventional TC; (ii) the maximum error due to inhomogeneity of the conventional type K TC is 10.75 °C; (iii) the maximum error due to inhomogeneity of the TCTF is below 0.2 °C.

## 1. Introduction

Instrumentation and measurements play a very important role in the modern economy [1]. The accuracy of measurements predetermines the quality of the production [1] and its reliability [2,3,4], safety [5], and service lifespan [2]. Modern industry and scientific research requires even better and more-accurate sensors, data acquisition units, and systems [6]. Recent improvements often apply artificial intelligence [7]. In recent times, the concept of the Internet of Things [8] has opened many new possibilities. For instance, it allows making systems global [8] and updating them with almost no efforts [8]. The main attention in this field of scientific studies is focused on the issues of the safety and security of data transfer [9]. However, we believe the accuracy of sensors is the key problem in this field. Sometimes this problem cannot be solved with the conventional measures, so new materials for sensors and sensors of new design appear.

The metrological procedures are estimated to constitute of 4% of the GDP in developed countries [1]. Temperature measurements are one of the most frequent measurements [10]. In spite of many techniques and sensors [10] for measuring temperature, the thermocouple (TC), in particular the base metal TC, is the most widely used sensor to measure temperature within the range 600–1200 °C [10,11]. However, the accuracy of TCs is often not adequate to satisfy modern demands [11,12,13,14,15,16]. The biggest error of the TC is error due to thermoelectric inhomogeneity acquired during long-term operation [12,17,18]. For instance, for the most common TC of the K type, this error can reach 11 °C [19,20] or 30 °C [13]. However, some studies report an even bigger error of over 100 °C [12].

Therefore, in the field of temperature measurements using base metal TCs, studies of error due to acquired thermoelectric inhomogeneity and the effective methods and means for its mitigation represents the most relevant problem.

## 2. State of the Art

The correction of the TC error by calibration to determine the real conversion function (CF) before operation [14] and its subsequent periodical calibration during operation is ineffective due to the significant impact of degradation of TC legs in the process of long-term measurement of high temperatures on the CF [21]. Degradation processes in TC legs cause two interconnected errors [14,15]:

1. Error due to drift of the CF of a TC. It is a gradual change in the developed thermal electromotive force (thermo-emf) in time in a constant temperature field along the TC legs. This error appears because of the degradation of TC legs under the influence of temperature and time of their operation.
2. Error due to acquired thermoelectric inhomogeneity. It is a change in the developed thermo-emf when the temperature distribution along TC legs changes (i.e., the temperature field along the TC changes), even for constant temperatures of the measuring and reference junctions. This error appears because of the degradation of the TC legs and changes in the temperature field along them.

Since the degradation of the TC legs is the cause of both errors, they can both be considered as different manifestations of the same phenomenon. So, the analysis carried out in [15] of the methods [15,16] for correcting the error due to TC drift showed their effectiveness only in a stable temperature field.

Concerning error due to inhomogeneity of TC legs, it was first mentioned in [17] more than a century ago, but the problem of inhomogeneity is still acute [12,15,18,19,20]. It is shown that this phenomenon has the greatest influence on the error of the temperature measurements using the TC. However, only error due to the initial inhomogeneity of the TC was investigated there. Improvement of the manufacturing process of the TC wire reduced this error to 1–1.5 °C. But, in [15,18,19], it was shown that during the long-term measurement of high temperatures, acquired thermoelectric inhomogeneity of the TC legs appears. It is caused by the fact that the degradation of TC legs runs much faster at high temperatures [21,22,23]. The principle of TC operation requires a temperature difference between the measuring and reference junctions [10]. Therefore, for the TC after several hours of operation, the dependence of the developed thermo-emf on the temperature field appears along its legs. As was mentioned above, for the most common TC of the K type, error due to acquired thermoelectric inhomogeneity can reach 11 °C [19], 30 °C [13], or even more than 100 °C [12]. Therefore, in [13], it was concluded that it is not reliable to use the results of the calibration of a TC in a laboratory furnace to correct its error under operating conditions. It should also be noted that error due to inhomogeneity is prone to all types of TC [12,24], not only to the type K TC or the base metal TCs.

However, the conclusion drawn in [13] was premature. In [15], the idea to use the correction obtained in the laboratory furnace was considered to compute the correction that can be used under operation conditions. However, this method was based on experimental studies, and the resulting correction did not take into account the individual deviations of the CF of a certain TC. In [15], the method for correcting error due to acquired thermoelectric inhomogeneity was also considered, which involved the calculation of an individual correction in a certain temperature field using a neural network trained on the calibration of the TC in several different temperature fields. The method provided high efficiency. It reduces the magnitude of error due to acquired thermoelectric inhomogeneity of TCs by four–five times with one-time short-term changes in the temperature field along the TC legs. The disadvantage of the method is its high laboriousness because of the need for experimental studies of the TC error in seven–nine temperature fields during each calibration.

**Figure 1:**
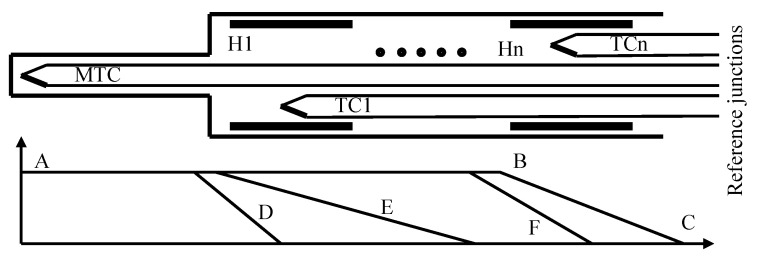
The concept of the TCTF.

The method of compulsory stabilization of the temperature field along the TC legs was proposed in [25,26,27]. This method of stabilization of the temperature field along the TC legs requires a new design of the TC-based sensor. This sensor was proposed in [25,26,27] and called the thermocouple with controlled temperature field (TCTF). The concept of this sensor is given in Figure 1 [26]. The main thermocouple (MTC) measures the temperature of an object. The subsystems to control the temperature field are located along the legs of the MTC. Each section consists of a heater (H1…Hn) and a corresponding thermocouple (TC1…TCn). All reference junctions of all TCs are wired to the measuring system, but this is not given in Figure 1 for the sake of simplicity. The temperature fields are given in the bottom of Figure 1. ABC is the temperature field set and maintained by the abovementioned control heaters H1…Hn. So, even if the temperature field of the object varies within the area limited by the temperature fields D…F, field ABC stays constant. According to [14,15,25,26,27], in such a stable temperature field, error due to acquired inhomogeneity of the MTC cannot manifest itself.

Forced stabilization is implemented using a set of temperature-control subsystems. The sensors of these subsystems (for example, the K type TCs, are of the same type as the MTC) and the corresponding heaters are located along the MTC [25,26,27]. Such a set of control subsystems, each of which maintains a preset temperature in its zone, allows for creating and maintaining a preset temperature field along the MTC legs. The latest studies revealed it is reasonable to physically implement the TCTF as a multi-zone tubular furnace placed in the wall of the measuring object, into which a conventional TC in a conventional thermowell is inserted [26,27]. In this case, the MTC can be substituted after reaching considerable degradation and the furnace can be used for a longer time.

It should be noted that error due to inhomogeneity of the TCTF still remains. It is caused by its imperfection, that is, the influence of changes in the temperature field of the measured object on the thermo-emf of the MTC, despite the stabilization of the temperature field along its legs.

The goal of this paper is to model the temperature measurement error caused by thermoelectric inhomogeneity acquired during the operation of both the TCTF and the conventional TC as well as to compare the results and determine the conditions when the use of the TCTF is reasonable.

## 3. The Technique of Modelling Error Due to Inhomogeneity

The theoretical basis of the developed technique for an indirect assessment of error due to inhomogeneity can be the conclusion drawn in [14]—errors due to acquired inhomogeneity and due drift of the CF of the TC are the consequences of the same phenomenon, such as the degradation of the TC legs. Therefore, the results of the experimental studies of TC drift under high operating temperatures can be used for estimating error due to inhomogeneity. The best results of studies of TC drift for the type K TCs are given in [22]. The main requirement for fitting functions of error due to drift in this case is their simplicity, differentiability, and continuity at an arbitrary point. The error of fit is less important.

According to [14], in TCs operated in stationary thermal aggregates, the thermo-emf, developed by each TC section ΔE, mainly depends on three variables—the operating temperature $t_{e}$, operating time $\tau_{e}$, and its instantaneous temperature $t_{d}$ (the temperature at which the section is at the instant of measurement). This can be given as follows: $\Delta E=f(t_{e},\tau_{e},t_{d})$. However, it is difficult to fit such a function because even the number of experimental data points in [22] is insufficient for such a fit. Therefore, in [28], it was suggested to fit the error for a fixed operating time. Then. it is reasonable to represent the function as a product of two functions of one variable as follows: $\Delta E=k\cdot f(t_{e})\cdot \varphi (t_{d})$, where the functions $f(t_{e})$, $\varphi (t_{d})$ are polynomials without intercepts, i.e., they are zero when either $t_{e}=0$ or $t_{d}=0$, respectively. These assumptions allow us to have enough data from [22] to fit the function of drift for the type K TC.

In the first stage, we fit the drift of the type K TC when the temperature of the measuring junction is 800 °C. According to [22], the drift of chromel $\Delta E_{X}^{1000}$ and alumel $\Delta E_{A}^{1000}$ legs after 1000 operating hours can be fitted by the functions as follows [28]:

(1) $$\Delta E_{X}^{1000}=0.035\sqrt{t_{e}}(-4.6\cdot 10^{-7}t_{d}^{3}+0.275\cdot 10^{-3}t_{d}^{2}+0.213\cdot t_{d})\mu V$$

(2) $$\Delta E_{A}^{1000}=0.035\sqrt{t_{e}}(-4\cdot 10^{-9}t_{d}^{4}+0.71\cdot 10^{-5}t_{d}^{3}-0.38\cdot 10^{-2}t_{d}^{2}+0.715\cdot t_{d})\mu V$$

**Figure 2:**
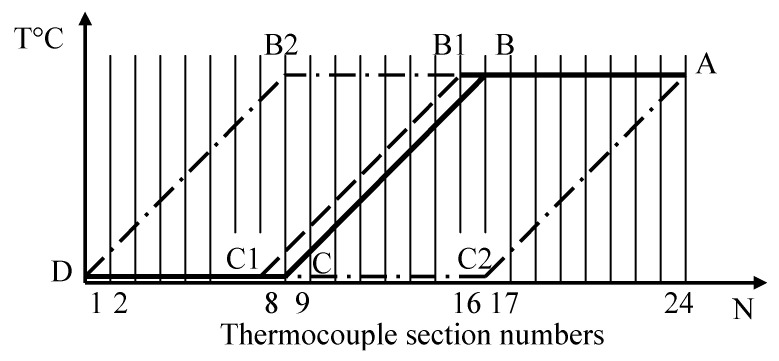
Division of electrodes of an inhomogeneous TC into sections.

To estimate error due to acquired thermoelectric inhomogeneity, we split both TC legs, similarly to [28], into 24 identical homogeneous sections (Figure 2), and set the following assumptions:

1. The temperature of the reference junctions of the TC is 0 °C. At this temperature, the TC sections from 1 to 8 are operated.
2. The temperature of the measuring junction of the TC is 800 °C. At this operating temperature, the TC sections from 17 to 24 are operated.
3. The sections of TC from 9 to 16 are operated in the zone of the temperature gradient.
4. The temperatures of sections can change within the range from 0 °C to 800 °C. The temperature drop across the *i*-th section is as follows: $\Delta t=t_{i}-t_{i-1}=100\;{}^{\circ}C$.
5. The sections of the TC are homogeneous along their length (i.e., there is no error due to acquired heterogeneity within each section).
6. The operating temperature $t_{e}$ of each TC section is considered the average temperature of temperatures of its ends before the change in temperature field.
7. The instantaneous temperature $t_{d}$ of the TC section is considered the average temperature of the ends of each section after the change in temperature field.

The error due to acquired inhomogeneity of the TC legs is equal to the change in the thermo-emf developed by these legs, for example, when the temperature field changes from A, B, C, D to A, B1, C1, D. According to [14,15], this is because the change in specific thermo-emf of sections (i.e., CF) from the nominal one depends on temperatures of operation of sections, that is, each section has its own specific thermo-emf, which depends on its operating temperature. The temperature drops across the TC sections after a change in the temperature field, which also plays a considerable role, because if the temperature drop equals zero in this particular section, according to the Seebeck law [10], it does not develop thermo-emf. This leads to different contributions to the total emf of various sections. To model this, at the first stage, we calculate the deviation of the CF from the nominal one for the sections of the chromel $\Delta E_{X}$ and alumel $\Delta E_{A}$ legs of the TC in the temperature field of operation (before the change in the temperature field from A, B, C, D to A, B1, C1, D), that is, under the condition $t_{di}=t_{ei}$. In other words, the temperature of operation and instantaneous temperature of each section of the TC are equal. We can calculate the deviations in the developed thermo-emf for both legs $\Delta E_{X}$ and $\Delta E_{A}$ using the Kirchhoff voltage law, according to the following formula [20,28]:

(3) $$\Delta E=\sum_{i=1}^{24}\Delta E_{i}=\sum_{i=1}^{24}\Delta e_{i}(t_{ei},t_{di})\cdot \Delta t_{i}$$

where $\Delta E_{i}$—the deviation of the developed thermo-emf from the nominal one for the *i*-th section of the corresponding TC leg; $t_{ei},t_{di}$—the temperatures of operation and instantaneous temperature of the *i*-th section of the TC leg, respectively; $\Delta e_{i}(t_{ei},t_{di})$—the deviation of specific thermo-emf of the *i*-th section of the TC leg; and $\Delta t_{i}$—the temperature difference between the beginning and the end of each TC leg (temperature drop across each section). According to the abovementioned assumptions, $\Delta t_{i}=100\;{}^{\circ}C$.

The drift of the specific thermo-emf $\Delta e_{i}(t_{ei},t_{di})$ for each TC section can be derived from (1) and (2) as partial derivatives with respect to instantaneous temperature $t_{d}$. For the chromel and alumel legs, we get the following expressions:

(4) $$\Delta e_{X}^{1000}=0.035\sqrt{t_{e}}(-13.8\cdot 10^{-7}\cdot t_{d}^{2}+0.55\cdot 10^{-3}\cdot t_{d}+0.213)\mu V/{}^{\circ}C$$

(5) $$\Delta e_{A}^{1000}=0.035\sqrt{t_{e}}(-16\cdot 10^{-9}\cdot t_{d}^{3}+2.73\cdot 10^{-5}\cdot t_{d}^{2}-0.76\cdot 10^{-2}\cdot t_{d}+0.715)\mu V/{}^{\circ}C$$

At the second stage, we calculate the deviation of the CF of chromel $\Delta E_{X}$ and alumel $\Delta E_{A}$ TC legs from the nominal one after the change in the temperature field from A, B, C, D to A, B1, C1, D, that is, when $t_{di}\neq t_{ei}$ (the temperatures $t_{di}$ of the TC sections from 8 to 16 were changed, and the temperatures of all other sections remain constant). We can calculate the deviation $\Delta E_{X}$ and $\Delta E_{A}$ according to (3), by plugging the corresponding value $t_{di}$ in (4) and (5), respectively. According to the definition given above, the difference in the deviations of the CFs of the chromel $\Delta E_{X}$ and alumel $\Delta E_{A}$ TC legs from the nominal one, obtained at the first and second stages (i.e., before and after the change in temperature field), is equal to the error due to thermoelectric inhomogeneity acquired during operation.

If we repeat the second stage of calculations for the fields corresponding to the displacement of the zone of temperature gradient from section 1 (filed A, B2, D) to section 17 (field A, C2, D), we get the dependence of TC error due to acquired inhomogeneity when its immersion is changed.

It should be noted, in field A, B2, D, sections 1…8, which develop thermo-emf, are operated at 0 °C, that is, according to [23], they have not undergone any degradation. In this case, we get the initial CF of the TC (i.e., without drift). For the field A, C2, D, sections 17…24, which develop thermo-emf, are operated at 800 °C, that is, at the maximum temperature. According to [22], the effect of the temperature of stable operation $t_{ei}$ on the degradation of the TC legs is crucial. The rule of thumb is the higher the temperature of stable operation, the bigger the degradation and, therefore, the bigger the error. Thus, the TC error for field A, C2, D will be maximum; therefore, the difference between the developed thermo-emf by the chromel $\Delta E_{X}$ and alumel $\Delta E_{A}$ TC legs in temperature fields A, B2, D and A, C2, D corresponds to the maximum error due to TC drift. At the same time, the same difference corresponds to the maximum error due to acquired thermoelectric inhomogeneity. Thus, the conclusion made in [14,15] about the equality of the maximum errors due to drift and due to acquired thermoelectric inhomogeneity is confirmed.

However, if one calculates the value of drifts for the chromel $\Delta E_{X}$ and alumel $\Delta E_{A}$ TC legs in such a way, it will turn out that their values do not correspond to experimental studies [22], which are the basis for models (1) and (2). This is due to the fact that traditional studies of error due to drift are carried out in a stable temperature field, that is, in temperature field A, B, C, D (see Figure 1). Then, the total change in the developed thermo-emf is developed both by sections 15 and 16, which, according to [22], underwent degradation maximally (because the rate of degradation strongly depends on operating temperature [22]), and by sections 8 and 9, which underwent very weak degradation because of the low operating temperature [23]. That is why the values of drift obtained in experimental studies are significantly lower than the maximum values.

To match the values of errors due to drift and due to acquired inhomogeneity, we use the similarity of the dependence of the drift curves of the legs’ CF on the operating time for different temperatures, which is obvious from the data given in [22]. Therefore, we introduce the proportionality coefficients $K_{P}$, equal to the ratio of the calculated value of maximum error due to acquired inhomogeneity $\Delta E_{NEOD}$ of a certain leg for the maximum operating temperature and the corresponding operating time to the experimentally determined in [22] error due to drift $\Delta E_{DR}^{MAX}$.

(6) $$K_{P}=\Delta E_{NEOD}/\Delta E_{DR}^{MAX}$$

The values obtained in the calculation according to Formulas (4) and (5) should be divided by the appropriate $K_{P}$ and then the error values will match the results of experimental studies [22].

The abovementioned technique allows us to estimate error due to acquired inhomogeneity of the conventional TC. It should be adapted for the TCTF. As mentioned above, the temperature-control subsystems placed along the MTC legs counteract the change in the temperature field along its legs, regardless of the change in the external temperature field. Therefore, this is carried out at the second stage, when calculating the developed thermo-emf of the chromel and alumel legs and their deviations $\Delta E_{X}$ and $\Delta E_{A}$ from the nominal CF after the change in the temperature field. For the TCTF, the condition $t_{di}\approx t_{ei}$ always holds, contrary to the conventional TC. However, then, the temperature difference $\Delta t_{i}=t_{di}-t_{ei}$ is still not equal to zero, which causes residual error due to inhomogeneity. This temperature difference is determined, according to [25,26,29], by the influence of the external temperature field on the internal one and the error of controlling the temperature field.

The influence of the external temperature field on the internal one $\Delta t_{P}$ for the TCTF is determined by multiplying the change in external temperature $\Delta t_{Z}$ by the penetration coefficient $K_{REAL}$ determined in [29]. For the prototype of the TCTF [29], the penetration coefficient is $K_{REAL}\leq 0.04$.

The error of control of the temperature field $\Delta t_{K}$ studied in [29,30] for the prototype reaches 1.3 °C. It should be noted that the error $\Delta t_{K}$ is random. It is superimposed on the error $\Delta t_{P}$ as a high-frequency noise. Then, when determining residual error due to acquired inhomogeneity of the section, the value of instantaneous temperature $t_{di}$ can be computed from the formula as follows:

(7) $$t_{di}=t_{ei}\pm 2\Delta t_{K}\pm \Delta t_{P}$$

We are intending to estimate the maximum residual error due to inhomogeneity of the TCTF, that is why, when calculating instantaneous temperatures of, for example, even sections, we assume $t_{di}=t_{ei}+2\Delta t_{K}+\Delta t_{P}$ to be substituted in Formulas (4) and (5), and for odd sections, $t_{di}=t_{ei}-2\Delta t_{K}-\Delta t_{P}$. In this case, the maximum residual error of the TCTF due to acquired thermoelectric inhomogeneity can be determined.

## 4. The Results of the Modelling of Error Due to Inhomogeneity

According to the abovementioned technique, described in Section 3, error due to acquired thermoelectric inhomogeneity when the external temperature field changes, both for chromel and alumel legs of the conventional TC alone, for the whole conventional TC, and for the TCTF when measuring the temperature of 800 °C for 1000 h, is estimated in this section.

**Figure 3:**
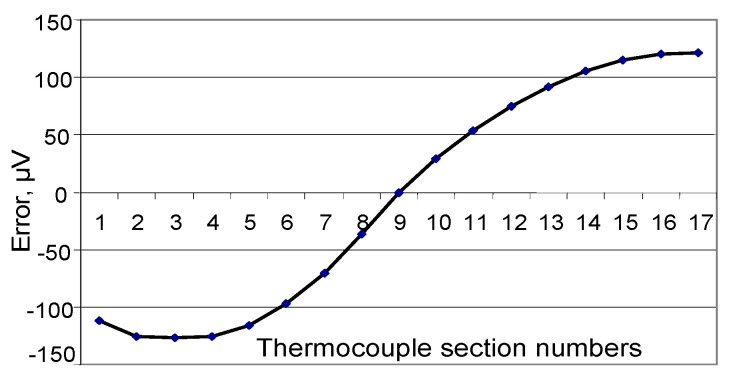
Error due to acquired inhomogeneity of the chromel leg of the conventional TC.

**Figure 4:**
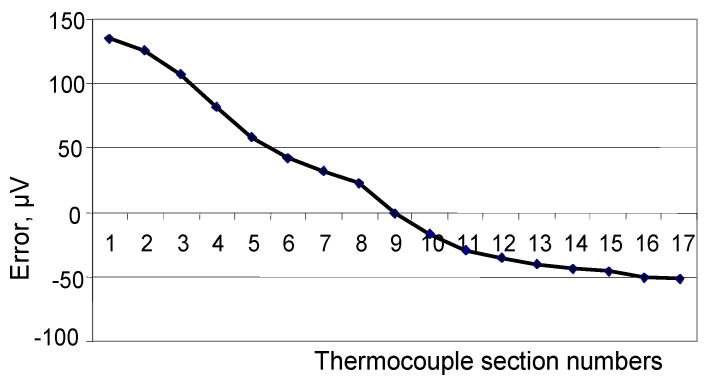
Error due to acquired inhomogeneity of the alumel leg of the conventional TC.

**Figure 5:**
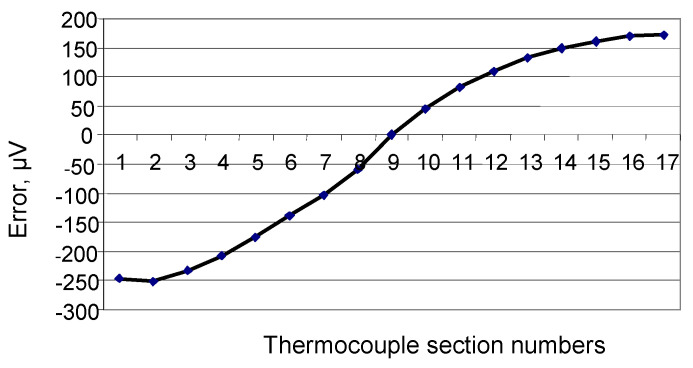
Error due to acquired inhomogeneity of the conventional type K TC.

Figure 3, Figure 4 and Figure 5 show the dependence of error due to acquired inhomogeneity on changes in the temperature field for the chromel and alumel legs, as well as the conventional type K TC, respectively. Proportionality coefficients $K_{P}$ determined according to (6) are equal to 2.11 for chromel and 1.39 for alumel. The peak-to-peak amplitude, i.e., the maximum difference between the biggest and smallest developed thermo-emfs, determined from Figure 3, Figure 4 and Figure 5, can be defined as error due to inhomogeneity. In Figure 3 the maximum value is 124 μV, the minimum value is −126 μV, and the difference reaches 250 μV for the chromel leg. For the alumen leg in Figure 4, the maximum value is 139 μV, the minimum value is −51 μV, and the difference reaches 190 μV. For the whole conventional TC, we can determine from Figure 5 the maximum value of 178 μV, the minimum value of −252 μV, and the difference of 430 μV. The last value of change in the thermo-emf corresponds to error of the conventional type K TC due to acquired inhomogeneity. The sensitivity of the type K TC is about 40 μV/°C. Thus, error due to inhomogeneity for the whole conventional type K TC can be estimated as

(8) $$\frac{430\;\mu V}{40\;\mu V/{}^{\circ}C\;}=10.75\;{}^{\circ}C.\;$$

**Figure 6:**
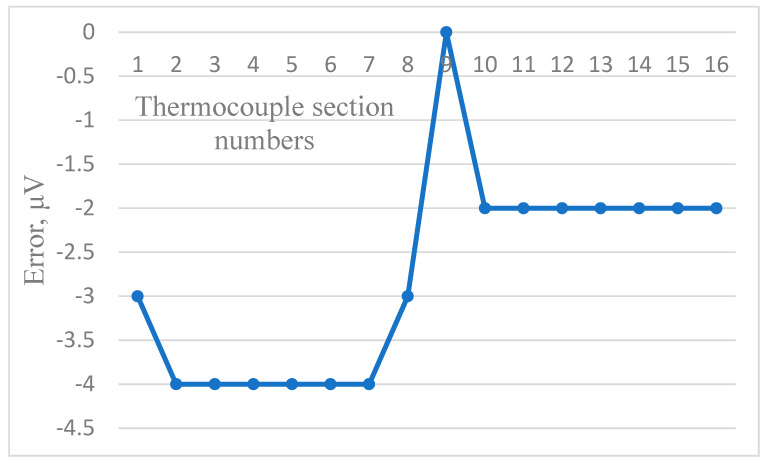
Residual error due to inhomogeneity of the chromel leg of the MTC.

**Figure 7:**
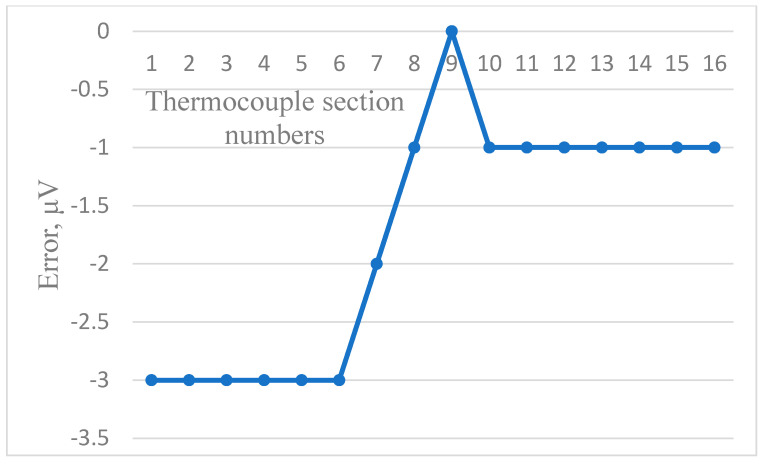
Residual error due to inhomogeneity of the alumel leg of the MTC.

**Figure 8:**
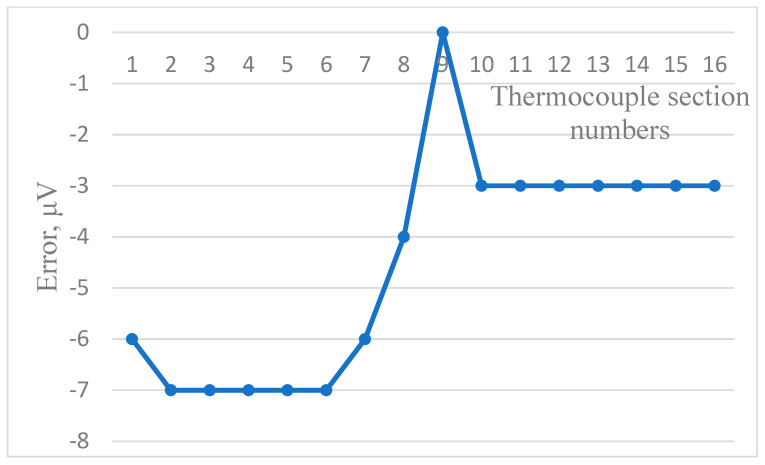
Residual error due to inhomogeneity of the whole MTC.

Figure 6, Figure 7 and Figure 8 show the dependence of residual error due to inhomogeneity of the chromel and alumel legs of the MTC, as well as for the whole MTC, respectively. The maximum residual error can be computed in the same way as in Figure 3, Figure 4 and Figure 5. As can be seen from the figures, the maximum error due to inhomogeneity of the chromel, alumel legs and the whole MTC is 4, 3, and 7 μV, respectively. The latter value of thermal electromotive force corresponds to residual error due to inhomogeneity and is approximately equal to 0.18 °C for the TCTF.

**Figure 9:**
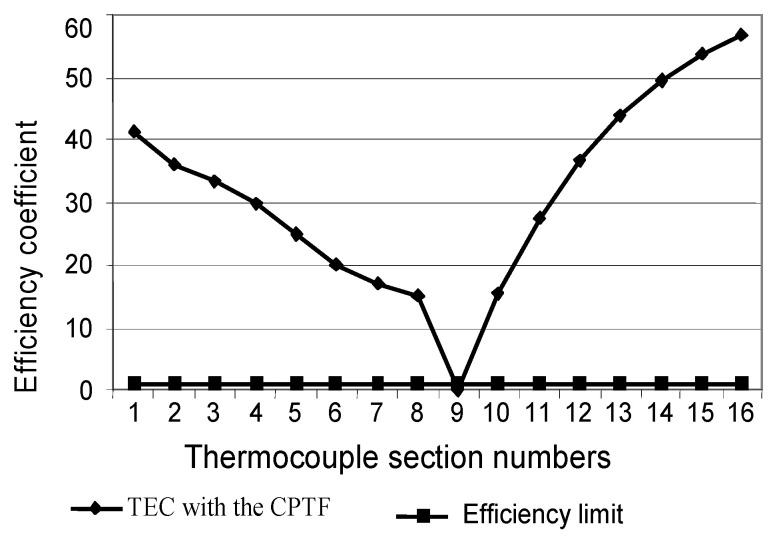
Efficiency coefficient vs. change in the temperature field.

However, despite the high efficiency of compensation of MTC error due to acquired inhomogeneity in the composition of the TCTF, in a constant temperature field of the measurement object, this error is smaller for a conventional TC. This is because the error of controlling the temperature field in the TCTF is always present. Therefore, even with a strictly constant temperature field of the object, residual error due to inhomogeneity manifests itself. Therefore, it is reasonable to investigate the efficiency ratio $K_{EF}$ of the TCTF to determine the conditions when it is reasonable to use it. The coefficient $K_{EF}$ can be determined based on the ratio of errors $\Delta_{TEP}$ of the conventional TC to error $\Delta_{TCTF}$ for the TCTF under the same changes in the temperature field of the object. Dependence $K_{EF}$ versus change in the temperature field is shown in Figure 9. It can be seen from the figure that the TCTF provides high efficiency under considerable changes in the temperature field. The limit of the reasonability of use of the TCTF is the condition of $K_{EF}=1$. From Figure 9, it is difficult to determine the limit $K_{EF}=1$, so the corresponding limits are indicated in Table 1.

As can be seen from Table 1, the limits of the reasonable use of a conventional TC correspond to small changes in the temperature field. If the temperature changes exceed the limits given in Table 1, it is reasonable to use the TCTF, because it is effectively suppresses error due to inhomogeneity. Both Figure 9 and Table 1 confirm the high efficiency of the TCTF.

## 5. Conclusions

After analysing the research results presented in the article, the following conclusions can be drawn:

1. The maximum deviation of the CF from the nominal for the whole type K TC is 430 μV. The initial sensitivity of the type K TC is 40 μV/°C; thus, maximum temperature measurement error due to thermoelectric inhomogeneity of the conventional TC acquired during long-term operation can exceed 10 °C, which cannot be neglected.
2. The maximum sensitivity of both the conventional TC and the TCTF to changes in the temperature field of the measurement object is for small changes in the temperature field, which makes this error even more dangerous.
3. The efficiency of the TCTF is quite high—the maximum value of error due to acquired thermoelectric inhomogeneity of the legs of the MTC is reduced by several tens of times to 0.2 °C. The high efficiency of error compensation allows using all drift correction methods considered in [15] with high reliability.
4. The high efficiency of the TCTF was confirmed in the studies above. The bigger the changes in the temperature field of the measurement object, the higher the efficiency of the use of the TCTF. However, it is achieved due to the relatively large structural complexity of the proposed sensor [15,25,26].
5. The limit of effective use of the TCTF is determined by the changes in temperature along the TC legs. In this particular case, when the temperature changes exceed 7 °C, it is reasonable to use the TCTF, because its error due to inhomogeneity is less than that of the conventional type K TC.
6. On the other hand, in a stable temperature field of the measurement object, the TCTF has a greater error due to acquired inhomogeneity than the conventional TC. This is because of the influence of the error in the temperature-control system. The reduction in this error will expand the limits of the effective application of the TCTF.
7. The dependence of residual error due to acquired thermoelectric inhomogeneity of the MTC of the TCTF on its structural complexity for given changes in the temperature field of the object remains unexplored. It is necessary to create a theoretical basis for designing the TCTF to ensure the necessary accuracy of temperature measurement results under the given conditions.

## Figures and Tables

**Table 1:** Limits of changes in the temperature field of the measurement object for effective use of the conventional TC.

The Direction of Change in the Temperature Field	
Towards the Reference Junctions	Towards the Measuring Junction
7 °C	6.5 °C

## Data Availability

The data can be obtained from the corresponding author on request.
